# Driving Distance and Food Accessibility: A Geospatial Analysis of the Food Environment in the Navajo Nation and Border Towns

**DOI:** 10.3389/fnut.2022.904119

**Published:** 2022-07-07

**Authors:** Natalie Bennion, Alisha H. Redelfs, Lori Spruance, Shelby Benally, Chantel Sloan-Aagard

**Affiliations:** Public Health, Brigham Young University, Provo, UT, United States

**Keywords:** spatial epidemiology, food security, indigenous food access, food accessibility, food environment

## Abstract

The Navajo Nation, an area home to approximately 173,000 people in the southwest United States, experiences the highest rates of food insecurity in the United States and is classified as a food desert. The present study assessed the accessibility to food outlets (grocery stores, convenience stores, and restaurants) as measured by driving time on the Navajo Nation and in selected surrounding border towns. Food outlets located in neighboring border towns were examined using network analysis tools in ArcGIS Pro to calculate driving distance and examine the potential impact of driving time within the Navajo Nation on accessibility to nutritious foods. There were 14 grocery stores, 21 convenience stores, and 65 restaurants identified in the Navajo Nation using Mergent Intellect, a proprietary database, as compared to border towns which had a total of 542 grocery stores, 762 convenience stores, and 3,329 restaurants equaling a ratio of about 50:1 (grocery, 39:1; convenience, 36:1; restaurants, 51:1) when comparing food outlets nearby versus on the Navajo Nation. This ecological study presents a visual representation of driving time and food accessibility, revealing geographic areas within the Navajo Nation where access to border town food stores is sparse, and food insecurity may be elevated.

## Introduction

Food insecurity is an economic and social condition characterized by limited or uncertain access to adequate food (1–3). The United States Department of Agriculture (USDA) defines food insecurity as “the disruption of food intake or eating patterns because of lack of money and other resources” (2). Risk factors for food insecurity include income, employment, and race/ethnicity (1). In 2019, 10.5% of United States households were food insecure at some point during the year, with 4.1% (5.3 million households) with very low food security (“multiple indications of disrupted eating patterns and reduced food intake”) (2, 4). Individuals with food insecurity often worry about food running out, that it will go to waste, and that they will not be able to afford a balanced meal (5). Food insecurity is most prevalent among individuals residing in poverty and is associated with various adverse health outcomes, including obesity, depression, type 2 diabetes, cardiovascular disease, and other chronic conditions on the Navajo Nation (6–9). Children who experience food insecurity have been found to have additional adverse childhood developmental behavior outcomes, including inattention, depression, latent early cognitive development, decreased social skills, and poor academic performance (10–12). Pardilla et al. found that 76.7% of individuals residing in the Navajo Nation experience some level of food insecurity, the highest rate of food insecurity reported for racial or ethnic groups within the United States (13).

The USDA classifies the majority of the Navajo Nation as a food desert. A food desert is a location where residents are low income and have low access to healthy food (14). The 2019 American Community Survey shows that 23.0% of American Indian/Alaska Native (AI/AN) people live below the poverty level, nearly twice the national average (12.3%) (15). In addition to socioeconomics, other factors associated with food insecurity across the Navajo Nation include reduced farming due to toxins in the soil (e.g., arsenic); the built environment (poor road networks, sparse food stores); reduced access to affordable nutritious foods; high food costs; and psychosocial factors including limited food knowledge and low healthy eating self-efficacy (13, 16–20).

The solutions to the food desert are equally difficult since the rural and low-income nature of the Navajo Nation makes it difficult to garner investment from grocery stores. Food-purchasing contexts such as price, geographic proximity to food stores, food shelf life, and ease of preparation also influence food insecurity coping strategies (21). Access to healthy food on the Navajo Nation is also a social and environmental justice issue amplified by forced relocations, environmental pollutants, colonialism, and loss of culture (19, 22). Over the past century, homegrown foods and household gardening have also decreased, resulting in a decline in intake of fruits and vegetables, and a greater portion of calories coming from processed and commercially prepared foods (17, 23). Moreover, there continues to be a limited number of nutritious foods available at local trading posts, which are small stores often used as the primary sources of purchased food among residents (24–26).

**FIGURE 1:**
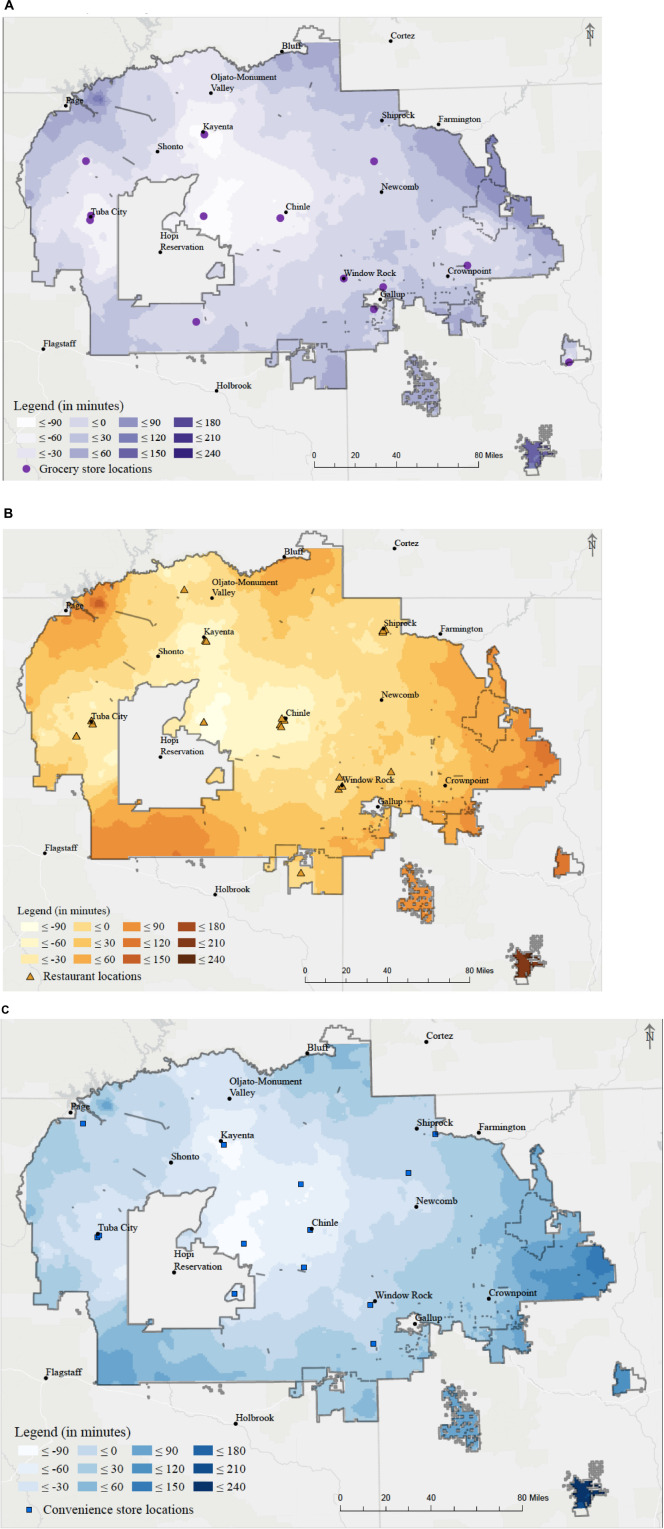
Description of the Navajo reservation. **(A)** Population density in the Navajo reservation by census tract, as persons per square mile. **(B)** Major road networks into and on the reservation.

The Navajo Nation covers a geographic area of 27,425 square miles that extends into Utah, New Mexico, and Arizona in the southwestern United States. Approximately 172,813 individuals reside in the Navajo Nation, 32% of whom are children younger than 19 years old (24). Though members of the Navajo tribe (“Navajo alone”) are represented in all parts of the United States, 47% reside within the Navajo Nation and have a population density of around 6.33 persons per square mile (Figure 1A). The Navajo Nation is organized by Agencies and Chapters. The most populated Chapters include Tuba City, Shiprock, and Chinle (Table 1). An additional 10% of Navajo alone reside in near or in surrounding border towns (16). “Border towns” are defined by the Navajo Epidemiology Center (NEC) as towns located outside the Navajo Nation with at least 500 Navajo or partially Navajo residents (16, 27). Most Navajo within the Navajo Nation live over 20 miles from the nearest supermarket and reside within a low-income census tract (5).

**TABLE 1:** Most populated chapters and driving distance to the nearest border town.

Major chapters	Total population	Distance to nearest border town^a^ (Border town, State)
Tuba City, AZ	9,265	74 miles (Page, AZ)
Shiprock, NM	9,126	28 miles (Farmington, AZ)
Chinle, AZ	8,005	92 miles (Gallup, NM)
Kayenta, AZ	6,211	76 miles (Bluff, UT)
Fort Defiance, AZ	5,856	31 miles (Gallup, NM)
St. Michaels, AZ/NM	5,643	28 miles (Gallup, NM)
Church Rock, NM	2,868	10 miles (Gallup, NM)
Pinon, AZ	1,084	104 miles (Holbrook, AZ)
Fruitland, NM	771	12 miles (Farmington, NM)
Many Farms, AZ	2,738	83 miles (Bluff, UT)
Crownpoint, NM	2,729	54 miles (Gallup, NM)

*^a^Border towns examined include Blanding UT, Cortez CO, Durango CO, Farmington NM, Gallup NM, Bloomfield NM, Grants NM, Aztec NM, Holbrook AZ, Flagstaff AZ, Page AZ, and Winslow AZ. Navajo Population Profile 2010 U.S. Census. https://www.nec.navajo-nsn.gov/Portals/0/Reports/NN2010PopulationProfile.pdf.*

Limited access to food has severe negative implications for health among residents of the Navajo Nation. Residents may shop for groceries only once or twice a month to reduce time and travel costs (28). Limited time and infrequent visits to grocery stores have multiple repercussions (2). Such practices affect supply and demand, causing fluctuations for store owners in terms of stocking nutritious foods and food spoilage rates based on when residents shop for food (at the start of the month, middle, or end) (1, 28, 29). Individuals often travel to border towns at the beginning of the month and over-purchase, leading to a sparsity of food by the end of the month (29). Finally, individuals often buy in bulk, with purchases reflecting energy-dense, processed, and pre-packaged food that can last for a long time without spoiling (4, 28, 29). These types of food are associated with obesity, diabetes mellitus, coronary heart disease, hypertension, and other chronic illnesses (30–32).

Though the factors that can influence food insecurity are well documented, there is a lack of research related to the Navajo Nation and the reliance of its residents on border towns to supplement food access. The purpose of this study was to create a visual representation of driving time and food accessibility. We hypothesized that it would reveal geographic areas within the Navajo Nation where access to border town food stores is sparse, making border food stores difficult to use as a supplement to the limited on-reservation access to nutritious foods. Additionally, the Navajo Nation is not a homogeneous food desert. There are areas of high food supply and high demand, areas of low food supply and low demand, and areas of low food supply but high demand. One of the main goals of this study is to identify and differentiate geographic regions across the Navajo Nation which can be prioritized due to imbalances in supply and demand. This study provides a visual and geographic depiction of the lack of food accessibility and aid public health, tribal, and government officials in addressing how border towns and Navajo Nation food access may influence efforts to reduce areas of food insecurity within the Navajo Nation.

## Materials and Methods

Our ecological study examined the driving time of individuals residing within the Navajo Nation to various food outlets (convenience stores, grocery stores, and restaurants) within and near the border as a measure of food access. We excluded locations and food outlets on the Hopi reservation, which is located within the borders of the Navajo Nation but is its own sovereign nation (33). Food accessibility was defined as the driving time, based on speed limits, between a digitally randomized point within the Navajo Nation and a specific food outlet address as determined by a network analysis and origin-destination cost matrix in ArcGIS Pro (34). A digitally randomized point for each potential resident (referenced as “origin”) was created due to unknown individual addresses of residents and to protect individual privacy (35). Border towns as defined by the NEC were used, including Blanding UT, Cortez CO, Durango CO, Farmington NM, Gallup NM, Bloomfield NM, Grants NM, Aztec NM, Holbrook AZ, Flagstaff AZ, Page AZ, and Winslow AZ (7, 36).

We identified food outlets using the proprietary Mergent Intellect database, which provides extensive information about public and private businesses by industry type, United States Census/Tigerline shapefiles from the United States Census Bureau, and ArcGIS road maps. North American Industry Classification System (NAICS) codes were used to classify each food outlet examined in this study. The NAICS was developed by the United States Economic Classification Policy Committee and is the standard used by federal statistical agencies in classifying business establishments (37). We used NAICS codes to identify the latitudes and longitudes of grocery stores, convenience stores, and restaurant locations.

Our main inclusion criterion for food stores and trading posts was the NAICS classification as either convenience stores, grocery stores, and/or restaurants. The NAICS code 445110 (grocery stores) included supermarkets and other grocery stores, excluding convenience stores. The NAICS code 445120 (convenience stores) included all convenience stores, trading posts, or food marts, which provide a limited line of goods that typically include milk, bread, soda, and snacks. Convenience stores with fuel pumps were excluded to maintain the focus on food-only outlets. Restaurants were identified using the NAICS code 72251. We used Google Earth to verify all food stores (grocery, convenience, and restaurant) individually.

We used the network analyst tool in ArcGIS Pro to examine driving time [trip-based travel impedance (38, 39)] from each food outlet store. Driving time was calculated using September 25, 2019, at 12:00 pm, to represent an average, mid-day driving schedule prior to the COVID-19 pandemic. Driving times analyzed were 30-, 60-, 90-, and 120-minutes away from each of the facilities. All food stores within the 120-min driving time were included in the study. Duplicates were deleted.

### Analysis

We generated 1,000 random points within the Navajo Nation boundary using the random point generator in ArcGIS Pro. Each random point represented an origin (or potential residence in the Navajo Nation). Destinations were defined as each of the food outlet stores. The ArcGIS Origin-Destination Cost Matrix (O.D. cost matrix) tool allows a maximum of 1,000 origins/destinations. There were less than 1,000 grocery stores and convenience stores included in the study, however, there were approximately 5,000 restaurants with a major percentage located in Albuquerque, NM. We decreased restaurant facilities to 1,000 (the ArcGIS limit) by only including Albuquerque restaurants west of the Rio Grande River and north of the I-40 interstate.

An O.D. cost matrix analysis was conducted to determine the minimum driving time from each origin to each destination. This was conducted for both food stores within the Navajo Nation and among border town locations. An output query was set to display only the one origin-destination connection with the shortest driving time. Minimum driving time as calculated by the O.D. cost matrix was used to compute an average driving time for residents residing within the Navajo Nation to any food outlet. We used Inverse Distance Weighting (IDW) to interpolate driving time and visually depict minimum driving time from within the Navajo Nation to the closest food outlet. The IDW used the minimum distance calculated in the O.D. cost matrix tool and created a smooth map representation of minimum driving time. We used the raster calculator tool to determine pockets of high food inaccessibility by subtracting Navajo Nation driving times from border town driving times. The outcome resulted in a raster map depicting low and high areas of driving time, shown in the figures.

## Results

**FIGURE 2:**
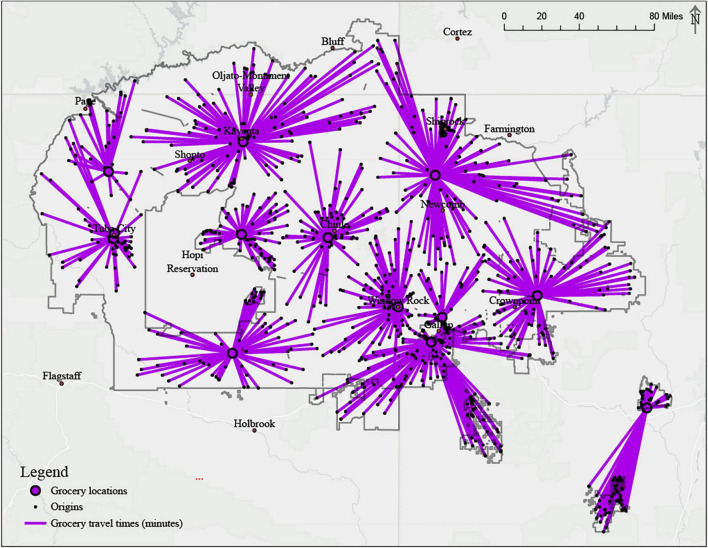
Origin-cost destination matrix tool; Grocery stores within the Navajo reservation.

The O.D. cost matrix displayed minimum driving time from each origin location within the Navajo Nation to food outlets on and off the Navajo Nation (Figure 2). There are three main towns within the Navajo Nation with a population density larger than 1,000 people per square mile. These towns include Fort Defiance AZ (population 5,856), Shiprock NM (population 9,126), and Tuba City AZ (population 9,265) (Table 1) (16). There are relatively few major roadways and networks within the Navajo Nation connecting towns and leading to towns outside the border (Figure 1B).

There were 14 grocery stores (0.08 per 1,000 people), 21 convenience stores (0.12 per 1,000), and 65 restaurants (0.38 per 1,000) identified in the Navajo Nation as compared to the border towns which had a total of 542 grocery stores (1.74 per 1,000), 762 convenience stores (3.51 per 1,000), and 3,329 restaurants (Table 2) (14.90 per 1,000), a ratio of about 50:1 (grocery, 39:1; convenience, 36:1; restaurants, 51:1) when comparing food outlets off versus on the Navajo Nation. The ratio per person (border towns/Navajo Nation) is 21.75 for grocery stores, 29.25 for convenience stores, and 39.21 for restaurants.

**TABLE 2:** Description of types of food outlets.

Types of food outlets	Border towns^a^ Description (N, example food outlets) (population 216,827)	Navajo reservation Description (N, example food outlets) (population 172,813)	Ratio food stores per person (Border towns/Navajo Nation)
Grocery stores	*N* = 542 Albertsons, Al’s mini-mart, Bashas’, Giant Food Stores, Pay and Save, and Safeway. (1.74 per 1,000 people)	*N* = 13 Bashas’, Westerners, T&R Market, City Market, Kaibeto Market, Tohajilee Food Market. (0.08 per 1,000 people)	21.75
Convenience stores (including trading posts)	*N* = 762 7-Eleven, Allsup’s convenience stores, Chevron food marts, Circle K stores, Expresses, Kum & Go stores, Mavericks, and Quick Stop Markets. (3.51 per 1,000 people)	*N* = 21 7-Eleven, Super K, Speedy’s convenience, Navajo Country Express, Tuba City Express, Blue Gap Trading Post, Tosto Trading Post, Round Rock Trading Post. (0.12 per 1,000 people)	29.25
Restaurants (including community stores)	*N* = 3,229 Subway, Pizza Hut, Village Inn, KFC, Starbucks, Arby’s, McDonalds, and Jack in the Box. (14.90 per 1,000 people)	*N* = 65 Subway, McDonald’s, Taco Bell, KFS, Burger King, Little Caesars Pizza, Sonic, China West Buffet, That’s a Burger, Pizza Edge. (0.38 per 1,000 people)	39.21

*^a^Border towns examined include Farmington, NM (population 44,967); Gallup, NM (population 21,637); Flagstaff, AZ (population 73,319); Page, AZ (population 7,551), Blanding UT (population 3,478), Cortez CO (population 8,729), Durango CO (population 19,413), Bloomfield NM (population 7,791), Grants NM (population 8,897), Aztec NM (6,467), Holbrook AZ (population 5,073), and Winslow AZ (population 9,505).*

On average, driving times to food stores within the Navajo Nation were shorter than driving times to food sources off the Navajo Nation. Travel time to grocery stores located within the Navajo Nation were 16.1 min faster than grocery stores in border towns. Travel time to restaurants located within the Navajo Nation were 1.7 min faster than restaurants in border towns. Travel time to convenience stores were 8.9 min faster than convenience stores in border towns. Traveling to grocery stores, convenience stores, and restaurants within the Navajo Nation had an average travel time of 50, 60, and 60 min, respectively (Table 3), assuming good traveling conditions and road maintenance.

**TABLE 3:** Driving time to food outlets.

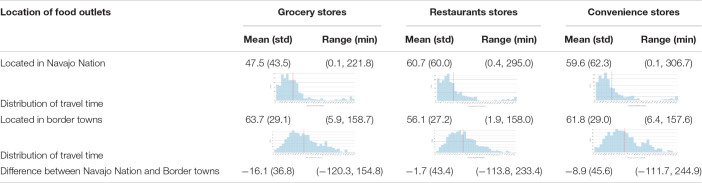

**FIGURE 3:**
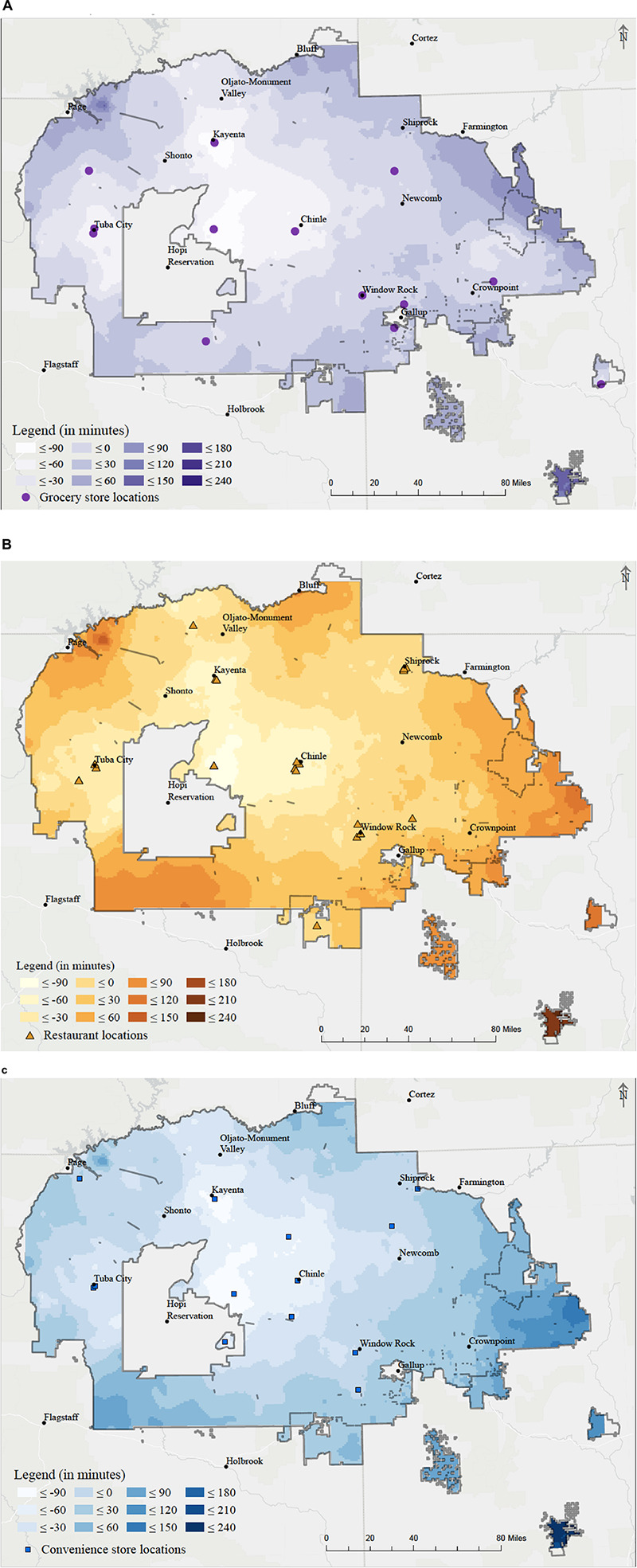
Food store driving time differences between on and off the Navajo reservation by minutes. **(A)** Grocery driving time differences. **(B)** Restaurant driving time differences. **(C)** Convenience store driving time differences.

We identified various areas where Navajo residents experienced very high driving times to food sources both within and outside the border. Rural towns near the middle of the Navajo Nation tended to have longer driving times to the 14 grocery stores on the Navajo Nation. A few towns within the Navajo Nation showed relatively no difference in travel time between traveling to food stores on the Navajo Nation or in border towns (Figure 3). Areas where travel time to food stores revealed no difference between border town locations and Navajo Nation locations were mainly towns with a population density of approximately 50 people per square mile, including Chinle AZ, Newcomb NM, and Crownpoint NM. Areas that had a faster travel time to food stores located in the Navajo Nation were those with a comparatively higher population density (500 people per square mile): Fort Defiance AZ, Kayenta AZ, and Shiprock NM.

Driving time analysis revealed a similar pattern among the three types of food outlet stores assessed (Supplementary Figure 1). Regardless of the type of food outlet analyzed, Navajo who resided south of Page AZ (Coppermine, Lechee), south Fort Defiance AZ (Window Rock AZ, St. Michaels AZ, Tse Bonito NM) and east of Shiprock NM (Kirtland NM, Fruitland) had the lowest amount of driving time to each food outlet. Individuals residing near Chinle NM, or the center of the Navajo Nation, had the longest travel time. Though geographically close to a border, towns in the northwestern part of the Navajo Nation had longer travel times, likely due to poor road networks and other geographical barriers.

## Discussion

Access to food outlets among those residing in the Navajo Nation is low due to the confluence of few food outlets on the Navajo Nation and long driving times to reach them. As stated by Sharkey, measures of accessibility in the context of food security are critical to target interventions to the areas of highest need (39). This study expands by using network analyst to identify rural and urban populations with low food accessibility.

The present study found a ratio of 50:1 in the number of food outlets off versus on the Navajo Nation in our study. The ratios per person range from approximately 21–39:1. The populations of the Navajo Nation and the Border towns are roughly comparable (173,000 vs. 216,000, respectively). The average one-way driving time to any of the three examined food outlets ranges between 55 and 65 min, with maximum driving times as long as 158 min (2 h 38 min). Several areas in the Navajo Nation, including some more populated towns such as Fort Defiance, show long driving times to food stores both within and outside the Navajo Nation. Furthermore, some central locations within the Navajo Nation have additional gas stations within the town that have limited fresh fruits and vegetables and majority processed foods (26). Nevertheless, those with the most extended driving times to food outlets are more likely to be those residing in rural areas; such communities already face numerous challenges with implementing health promotion initiatives and interventions compared to urban residents (40, 41). This is not surprising when we consider that 2/3 of the 14,000 roads within the Navajo Nation are not paved (42). The paved roads connect the larger towns within the Navajo Nation to border towns while the more rural locations often have only dirt roads that can be impassible when it rains or snows, creating further disparities in access among Navajo Nation residents living in rural areas and illustrating the heterogeneity of the Navajo Nation.

The present data are consistent with previous results showing the impact of driving on supply and demand accessibility to nutritious food. Residing in a location where food accessibility is low and where it takes as long as four or more hours to buy groceries is common among residents of the Navajo Nation (28). Additional driving-related challenges to food access include traveling expenses, limited access to vehicles, lack of public transportation, and time coordination with the long driving time to purchase their food. High driving time and low accessibility to food stores impact outcomes like diet quality and food choice (43, 44). Other factors that influence the distance individuals are willing to travel include prices of food products, spendable income available, and food knowledge (29, 43, 45). Individuals with poorer diet quality tend to drive longer distances to food stores and live in areas with lower residential property values than those with higher quality diets (44) which is consistent with what we know about the relationships between food insecurity, low income and residing in food deserts (13, 18). Our results and the existing literature provide convincing evidence that driving is a key influencer of access and food insecurity for a portion of the residents of the Navajo Nation.

Based on our findings, we would expect the residents living in the pockets within the Navajo Nation that have the longest driving times (near Chinle NM, central, and northwestern portions) to experience both less access to nutritious foods and higher costs for nutritious foods. This heterogeneous disparity is consistent with the literature related to choice patterns, food costs, and availability of nutritious foods within the Navajo Nation. Systematic review supports proximity, cost, limited availability of nutritious foods, consumer perceptions of food quality, and seasonal factors as common barriers to promoting healthy food options in American Indian reservations (45). Observation studies (National Environment Measures Surveys-Stores) confirm that healthier food costs more per unit than unhealthy food in the Navajo Nation (25) and data indicate that 64–70% of Navajo money or approximately $0.53–0.65 of every dollar is spent off the Navajo Nation, including money spent on food (36, 46). However, food shopping behavior is complex, meaning that individuals may bypass the nearest supermarket or travel longer distances to specific stores due to price, quality, and quantity of items, or current stock of certain items, such as fresh fruits and vegetables (43), and these patterns vary based on factors such as seasonality (29).

As in many populations, it is common for interventions among the Navajo Nation to focus on individual level interventions to improve health. The Community Outreach and Patient Empowerment (COPE) intervention, launched by the Centers for Disease Control and Prevention (CDC), is an individual intervention that improves health through use of community health representatives to improve patient care for medically underserved people living with chronic conditions, including diabetes, in the Navajo Nation (47). The COPE model using community health representatives was moderately successful at addressing the most vulnerable, but impact is limited by each. In practical terms, the rural nature of the Navajo Nation and the large distances involved may mean that those further from population centers are less likely to participate in such individual-level interventions. Interventions at the community, systems or policy levels [those with the greatest reach, tend to have the greatest long-term impact on population health (48)]. The Diné Food Sovereignty report supports this conclusion, as survey respondents, community-based solutions or interventions focused on empowering community members would have a longer lasting impact than purely governmental programs or individual-based interventions (46).

Furthermore, with the Healthy, Hunger-Free Kids Act (HHFKA) of 2010, the USDA established GIS mapping services of local food networks for Navajo communities and stakeholders. This initiative called the “Navajo Nation Food Access Navigation Program/Healthy Hunger-Free Kids Act” was designed to “enhance access to healthy nutritious food for all children for a healthier Diné Nation by promotion, preserving, and empowering Diné cultural values and traditional teachings through partnerships and collaboration with local, state, and governmental entities.” This program analyzed the food resources of schools and 36 Navajo communities. Findings reported that food and nutrition implementation efforts varied at schools and chapter communities, and barriers included limited outreach, lack of transportation (students, participants, and staff), limited food sources, and high participant turnover (short-staffed) (49). Our study provides additional knowledge and visual depictions of food resources and introduces border town supplementation for food access. It also builds on the conversation that systemic changes to the administration of food on the Navajo Nation and enhancement, promotion, or federal funding of community gardens may have a more sustainable impact on food insecurity among the Navajo Nation.

Our study supports the argument that we need to move beyond individual interventions (50) toward community- or system-level intervention to have a long-term effect on decreasing food insecurity. Those with the longest driving times to food outlets are more likely to be those residing in rural communities where communities already face numerous challenges with the implementation of health promotion initiatives and interventions compared to urban residents (40, 41). Several successful community and system-level interventions focus on increasing produce and food accessibility among the Navajo Nation (49, 51–54). For example, the Navajo Fruit and Vegetable Prescription Program (Navajo FVRx) aims to improve fruit and vegetable consumption by providing vouchers that can be redeemed at food stores within the Navajo Nation (51). The highlight of this program is that it is community-driven and culturally relevant, allowing community members and relevant stakeholders to change the design of the program depending on the needs and feedback from the community (51, 52). Other community- and systemic-level interventions are focused on education in schools and teaching children how to grow gardens at school to later implement at home (53). These programs aid at increasing community and individual efficacy by providing Navajo Nation residents with knowledge and tools to decrease the food insecurity levels regardless of whether food outlets are nearby. However, such programs do not to increase access by reducing the distance between Navajo Nation residents and food stores that have fruits and vegetables available.

Overall, our findings together with previous studies suggest a need for environmental change interventions to decrease barriers to access at the community level to address disparities in access to nutritious foods within the Navajo Nation. The Healthy Diné Nation Act in 2014 was one attempt to address this gap by making nutritious foods tax-exempt to encourage small stores to carry more fruits and vegetables (51). It would be important to consider how population-level interventions should vary depending on the degree to which each community has relative access to food sources (e.g., border town, on-reservation, or neither). Layering of tailored community and policy level interventions will be necessary to increase the availability of nutritious foods within existing stores in the Navajo Nation and address the existing health disparities.

Limitations of this study include being a cross-sectional, descriptive study. Driving times may differ significantly by season and time of day. Navajo Nation residents may potentially supplement their food intake with smaller food stores or mobile restaurants that were not included in our dataset. Further research is needed on how often individuals drive to Navajo Nation or border town food stores and what types of food are sold and most often purchased at the specific food outlets. This would give a more accurate representation of food insecurity among the Navajo Nation.

This study is one step toward filling the gap in the research about the reliance of residents of the Navajo Nation on border towns as a supplement to limited food access on the Navajo Nation. It has been argued that proximity to a given community resource is not the only consideration in its use. As such, next research steps may include the application of GPS data (cell phones) and newly developed utility-based accessibility models that consider attributes of the destination food store in lieu of only considering distance and driving time to provide a more granular understanding of the factors that influence choice and accessibility (55).

The use of spatial and origin-destination cost analyses allows public health officials, including members of the Navajo tribal council, Navajo Department of Health, the United States Bureau of Indian Affairs, and the United States Department of Health and Human Services *via* the Indian Health Service division to visually depict the lack of food access and high food insecurity among the Navajo Nation. This may provide opportunities to improve infrastructure through Healthy Food Financing Initiatives (HFFI) sponsored by the United States Department of Agriculture (56). One potential approach could use HFFI to fund a “corner store initiative” to retrofit existing retail locations to allow more nutritious foods to be carried while minimizing waste (26, 57). Additionally, findings from the present study may create policy opportunities including changes in zoning to allow for grocery stores to open in new areas, incentives to help groceries stores build in new locations, and further research devoted to assessing policy implications of new initiatives.

Food insecurity rates within the Navajo Nation are disproportionately high and are, at least in part, attributable to high poverty and unemployment rates and are exacerbated by poor access to nutritious foods due to a limited local availability, inadequate infrastructure, and long driving times. Reducing food insecurity will require improving the availability of affordable nutritious foods and decreasing driving time through evidence-based community-level practices and programs.

## Data Availability Statement

The data analyzed in this study is subject to the following licenses/restrictions: The primary database is proprietary, as stated in the article, but available through many libraries and universities. Requests to access these datasets should be directed to Mergent Intellect.

## Author Contributions

NB, LS, AR, SB, and CS-A: conceptualization, and writing – review and editing. NB: methodology and resources. NB and CS-A: formal analysis, writing – original draft preparation, and visualization. CS-A: supervision. All authors have read and agreed to the published version of the manuscript.

## Conflict of Interest

The authors declare that the research was conducted in the absence of any commercial or financial relationships that could be construed as a potential conflict of interest.

## Publisher’s Note

All claims expressed in this article are solely those of the authors and do not necessarily represent those of their affiliated organizations, or those of the publisher, the editors and the reviewers. Any product that may be evaluated in this article, or claim that may be made by its manufacturer, is not guaranteed or endorsed by the publisher.

## Supplementary Material

Supplementary Figure 1 Driving distances by location on and off the Navajo reservation in minutes. **(A)** Grocery driving distances within the Navajo reservation. **(B)** Restaurant driving distances within the Navajo reservation. **(C)** Convenience driving distances within the Navajo reservation. **(D)** Grocery driving distances in border towns. **(E)** Restaurant driving distances in border towns. **(F)** Convenience driving distances in border towns.
