# Supplementary material for: Driving Distance and Food Accessibility: A Geospatial Analysis of the Food Environment in the Navajo Nation and Border Towns
Source: Front Nutr. 2022 Jul 7;9:904119. doi: 10.3389/fnut.2022.904119 (PMC9301304; doi:10.3389/fnut.2022.904119)

Supplemental figures

Fig S1 Driving distances by location on and off the Navajo reservation in minutes (a) Grocery driving distances within the Navajo reservation (b) Restaurant driving distances within the Navajo reservation (c) Convenience driving distances within the Navajo reservation (d) Grocery driving distances in border towns (e) Restaurant driving distances in border towns (f) Convenience driving distances in border towns

(a) Grocery driving distances within the Navajo reservation

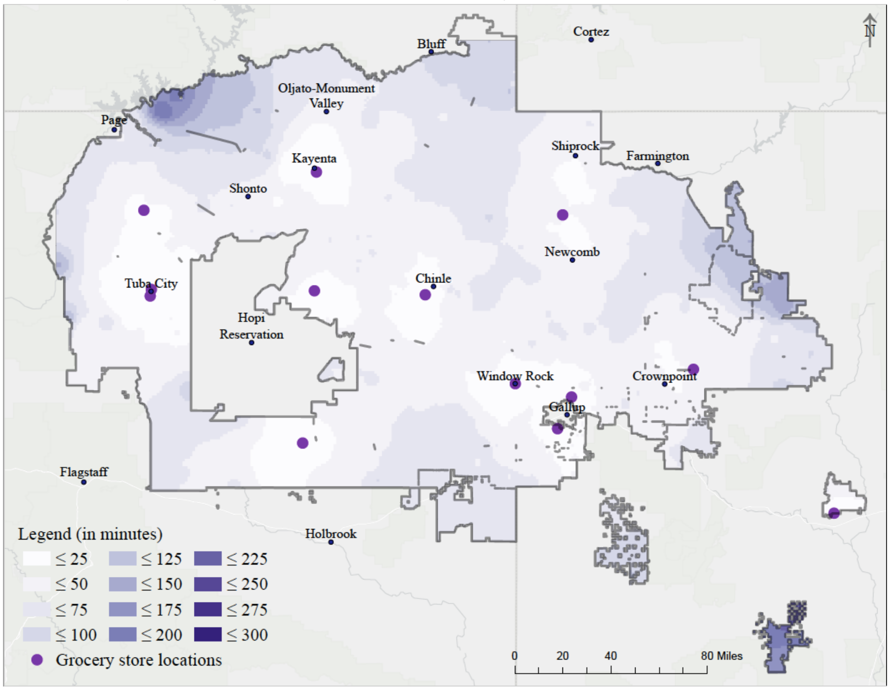

(b) Restaurant driving distances within the Navajo reservation

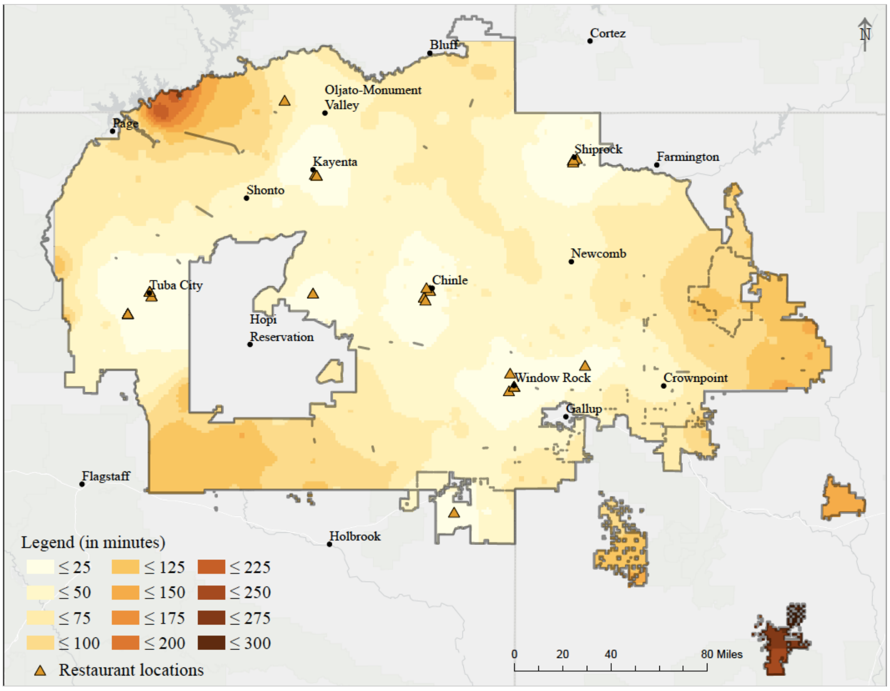

(c) Convenience driving distances within the Navajo reservation

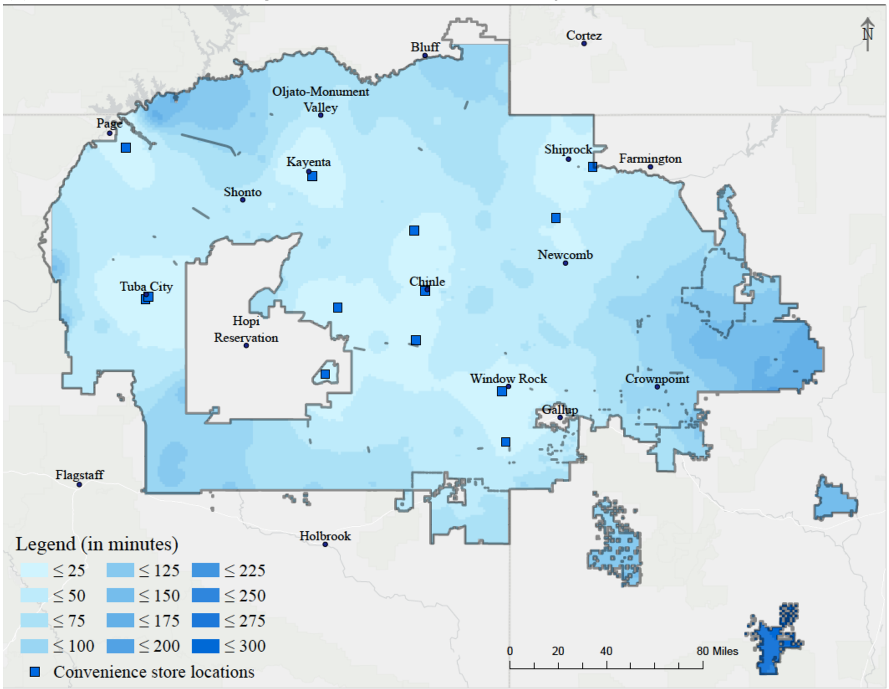

(d) Grocery driving distances in border towns

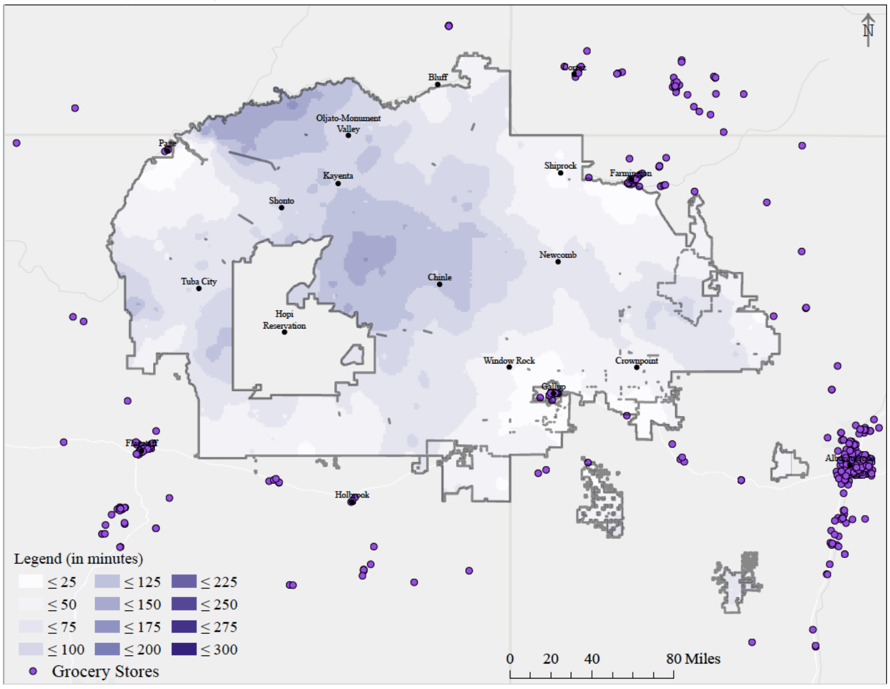

(e) Restaurant driving distances in border towns

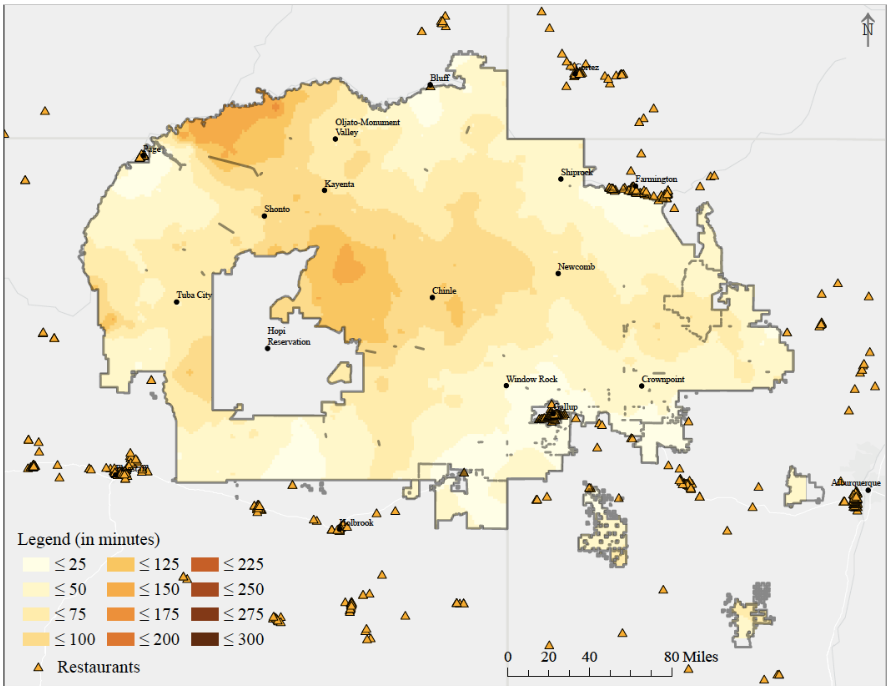

(f) Convenience driving distances in border towns

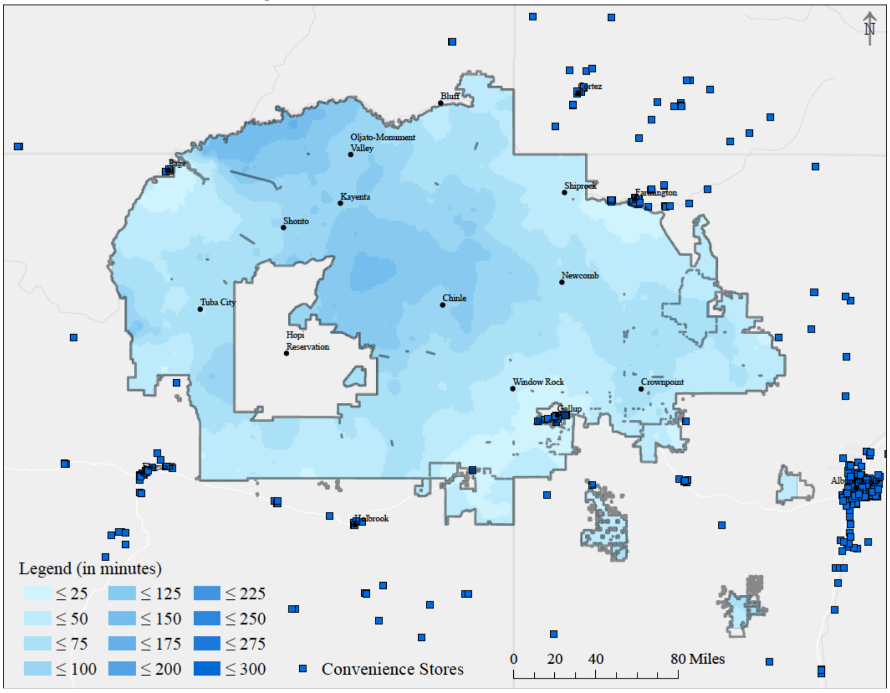

Supplement: Supplementary Figure 1 — Driving distances by location on and off the Navajo reservation in minutes. (A) Grocery driving distances within the Navajo reservation. (B) Restaurant driving distances within the Navajo reservation. (C) Convenience driving distances within the Navajo reservation. (D) Grocery driving distances in border towns. (E) Restaurant driving distances in border towns. (F) Convenience driving distances in border towns. [file Image_1.pdf]
